# Embracing the complexity of cooperation

**DOI:** 10.7554/eLife.108039

**Published:** 2025-07-15

**Authors:** Benjamin Allen

**Affiliations:** 1 Department of Mathematics, Emmanuel College Boston United States

**Keywords:** Price equation, Hamilton's rule, Queller's rule, kin selection, population genetics, relatedness, None

## Abstract

A theoretical framework for analyzing the evolution of nonlinear cooperative interactions is taking shape.

**Related research article** van Veelen M. 2025. The general version of Hamilton’s rule. *eLife* **14**:RP105065. doi: 10.7554/eLife.105065.

Nature is marked not only by struggles for survival, but also by remarkable feats of cooperation. From microbes to insects to humans, organisms work together in a variety of ways to gather and share resources, to build habitats, and to defend against common threats.

One explanation for the evolution of cooperation is shared genes. For example, if an individual saves the life of a close relative, the genes for this cooperative behavior may also be found in the relative’s genome, and could spread from there to future generations. In 1964, attempting to quantify this insight, the evolutionary biologist WD Hamilton proposed a now-famous rule: a cooperative behavior is favored by natural selection if *br* >*c*, where *b* is the benefit in fitness to the recipient, *r* is the relatedness of the recipient to the actor, and *c* is the cost in fitness to the actor (Hamilton, 1964).

Hamilton’s rule is remarkably simple, but applying it to real-world cooperation leads to thorny complications. In principle, the cost to the actor and the benefit to the recipient quantify how genetic predisposition towards a helpful trait affects the expected number of future offspring for the actor and recipient, respectively. Such effects cannot be measured empirically, since an organism’s reproductive success depends on an unknowable variety of events and factors. Moreover, real-world cooperation often involves multiple individuals, who may contribute in different ways and to different extents, with the effects of these contributions combining in nontrivial ways (Archetti et al., 2020). This calls into question whether real-world cooperation can ever be captured by one benefit and one cost parameter, as Hamilton’s rule requires.

The standard response to these difficulties is to apply linear regression (Queller, 1992; Gardner et al., 2011). In this approach, both the benefit and the cost are re-defined as slopes in a linear regression model, with genetic propensities to cooperate as explanatory variables, and offspring number as the response variable (Figure 1A). This method yields values of *b*, *r* and *c* such that *br* >*c* if and only if the cooperative trait increases over the course of a single generation. However, since the change in trait frequency is already known before applying linear regression, this approach does not yield any new predictions (Nowak et al., 2017). Moreover, if the effects of cooperation on fitness are nonlinear, the resulting values of *b* and *c* tell us little about why a particular behavior was selected, or what outcomes might be expected at different times or under different conditions.

**Figure 1.**
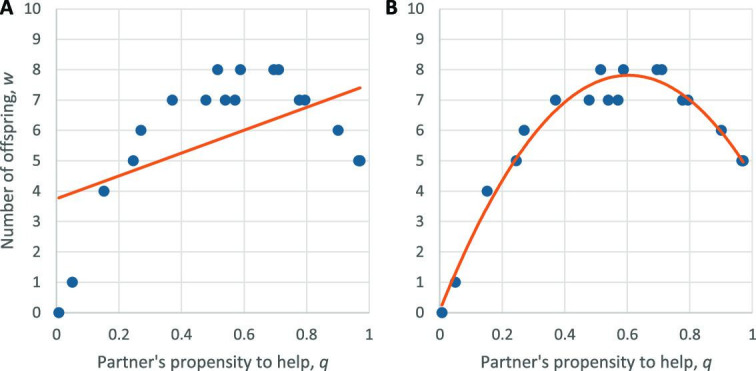
Linear and nonlinear regression models for the benefits of helping behavior. (**A**) A standard approach to quantifying the benefit $b$ and cost $c$ of cooperation is to apply a linear regression model of the form $w=w_{0}- cp+bq+\epsilon $, where $w$ is an individual’s offspring number, $w_{0}$ is a constant term, $p$ is the genetic propensity of the individual to cooperate, $q$ is the genetic propensity of the individual’s partner to cooperate, and $\epsilon $ is a residual term. Benefit and cost are then determined by applying least-squares regression to population data for a single generation. Here, for simplicity, we show hypothetical data for $w$ and $q$ only (blue points), ignoring the effects of $p$. In this scenario, offspring number peaks at an intermediate level of help. Fitting a linear model $w=w_{0}+bq+\epsilon $ (orange line) results in a “benefit” $b$ that sheds little light on the nature of the cooperative trait. (**B**) Fitting a quadratic model $w=w_{0}+b_{0,1}q+b_{0,2}q^{2}+\epsilon $ (orange curve), as van Veelen’s framework allows for, yields a more informative characterization of cooperation in this scenario.

Now, in eLife, Matthijs van Veelen of the University of Amsterdam reports how the regression method can be generalized to allow for arbitrary nonlinear relationships (van Veelen, 2025). While the idea of using nonlinear regression has been occasionally suggested before (Queller, 1985), van Veelen provides a general framework for applying it to any interaction between two organisms.

For example, if helping behavior has a quadratic effect on reproductive success, Hamilton’s rule can be rewritten to become *b*_0,1_*r*_0,1_ + *b*_0,2_*r*_0,2_>*c*, where *b*_0,1_ and *b*_0,2_ quantify the linear and quadratic effects of helping, and *r*_0,1_ and *r*_0,2_ quantify the linear and quadratic relatedness of the actor to the recipient (Figure 1B). Likewise, an interaction effect between the propensities of both parties to cooperate can be included by adding a term *b*_1,1_*r*_1,1_ to the left-hand side of the condition. Van Veelen’s framework allows for any number of terms of the form *b*_*k*,*l*_*r*_*k*,*l*_ to be added, where *r*_*k*,*l*_ quantifies a nonlinear genetic association in cooperativity levels, of order *k* in the recipient and order *l* in the actor, and *b*_*k*,*l*_ quantifies the effect of this association on reproductive success.

While van Veelen’s rule is more complex than Hamilton’s, it enables greater realism by allowing us to choose a model appropriate to the biological situation at hand. Like the linear regression version, van Veelen’s method does not predict the outcome of selection, which must be specified in advance. The advantage of van Veelen’s framework lies in characterizing selective change using parameters that meaningfully describe a given interaction and its relationship to reproductive success, without presupposing what form this relationship will take.

Van Veelen’s work underscores a growing appreciation of the role of nonlinear interactions within evolutionary biology, especially with regard to the evolution of cooperation. Classical evolutionary theory, dating back to the modern synthesis (Fisher, 1919), assumes that each gene has a specific effect on an organism’s survival and reproduction, with the effects of different genes adding together in linear fashion. Hamilton’s rule applies this linear thinking to cooperative behavior. But empirical studies show that real-world cooperation cannot be captured by simple linear models (Archetti et al., 2020). Van Veelen’s framework, along with other recent advances (Allen et al., 2024; Sheng et al., 2024), helps provide a theoretical foundation for analyzing the evolution of nonlinear cooperative interactions. Ultimately, these efforts bring us closer to a full understanding of how cooperation arises, from microbial communities to human societies.
